# High Frequency Sampling of TTL Pulses on a Raspberry Pi for Diffuse Correlation Spectroscopy Applications

**DOI:** 10.3390/s150819709

**Published:** 2015-08-12

**Authors:** Matthew Tivnan, Rajan Gurjar, David E. Wolf, Karthik Vishwanath

**Affiliations:** 1 Radiation Monitoring Devices Inc., 44 Hunt Street, Watertown, MA 02472, USA; E-Mails: tivnan.m@husky.neu.edu (M.T.); Rajan.Gurjar@ll.mit.edu (R.G.); dwolf@pendarmedical.com (D.E.W.); 2 Department of Electrical and Computer Engineering, Northeastern University, Boston, MA 02115, USA; 3 MIT Lincoln Laboratory, Lexington, MA 02421, USA; 4 Pendar Medical, Cambridge, MA 02138, USA; 5 Department of Physics, 500 E Spring Street, Miami University, Oxford, OH 45056, USA

**Keywords:** software autocorrelation, blood flow, Raspberry Pi, optical spectroscopy, coherent scattering, laser speckle

## Abstract

Diffuse Correlation Spectroscopy (DCS) is a well-established optical technique that has been used for non-invasive measurement of blood flow in tissues. Instrumentation for DCS includes a correlation device that computes the temporal intensity autocorrelation of a coherent laser source after it has undergone diffuse scattering through a turbid medium. Typically, the signal acquisition and its autocorrelation are performed by a correlation board. These boards have dedicated hardware to acquire and compute intensity autocorrelations of rapidly varying input signal and usually are quite expensive. Here we show that a Raspberry Pi minicomputer can acquire and store a rapidly varying time-signal with high fidelity. We show that this signal collected by a Raspberry Pi device can be processed numerically to yield intensity autocorrelations well suited for DCS applications. DCS measurements made using the Raspberry Pi device were compared to those acquired using a commercial hardware autocorrelation board to investigate the stability, performance, and accuracy of the data acquired in controlled experiments. This paper represents a first step toward lowering the instrumentation cost of a DCS system and may offer the potential to make DCS become more widely used in biomedical applications.

## 1. Introduction

### 1.1. Diffuse Correlation Spectroscopy

Diffuse Correlation Spectroscopy (DCS) is a non-invasive method that has been developed [1,2] over the last few decades and provides a powerful technology that can been used to monitor hemodynamic properties of biological tissue *in vivo* DCS has been used clinically to measure blood flow within various tissues including skeletal muscle [3,4,5] and the brain [6,7,8,9,10,11], and has been validated via comparison to standard clinical imaging modalities including MRI and ultrasound. Typically, a DCS system consists of a coherent near-infrared (NIR) laser source, a fiber optical probe containing the source and detector fibers, a fast avalanche photodiode detector (APD), and a correlation device that collects the temporal intensity output from the APD and performs the intensity autocorrelation.

The experimental implementation and the theoretical analysis of DCS have both previously been well described [12,13,14,15]. Briefly, the light incident from a laser source is coupled to the tissue via an optical fiber (typically 100–200 µm in diameter) and the light that emerges from the turbid sample (tissue) after interacting with it is detected using a single-mode fiber. The detection fiber typically carries the reflected (or transmitted) intensity from one-to-two speckles contained in the coherent input source. The distal end of the detection fiber is coupled to a fast APD detector that is made to operate in the photon counting (or Geiger) mode. The electronic output from the APD is a digital signal of Transistor-transistor logic (TTL) pulses. Each pulse represents the arrival of a photon packet at the detector, remitted from within the tissue after scattering interactions with the medium. A temporal sequence of detected photons is collected and used to compute the autocorrelation function of the signal using a correlation device. This intensity autocorrelation is related to the autocorrelation of the temporal electric field of the incident (coherent) photon field within the medium. Typically, the collection of the temporal output from the APD as well as the computing of its autocorrelation is done using a hardware correlator board. In this report, we demonstrate a proof-of-concept for the development of a data acquisition system to collect and process intensity autocorrelations for DCS applications using a low-cost Raspberry Pi minicomputer.

### 1.2. Raspberry Pi

The Raspberry Pi is a computing device with dimensions spanning a credit card (~3.4″ × 2.2″). It was developed by the Raspberry Pi Foundation as a low-cost system-on-a-chip (SoC) device for the study of basic computer science in schools [16]. The first generation board on the Raspberry Pi was built with a 700 MHz Advanced RISC Machines (ARM) processor. The main motherboard on the Raspberry Pi also contains several peripherals including general purpose input/output (GPIO) pins. The GPIO circuitry is designed to be able to read digital input signals between 0–3.3 V across its pins and is rated to operate up to a maximum rate of 125 MHz [17]. Thus, it provides a flexible means to rapidly sample digital signals that range between 0–3.3 V. The first generation Raspberry Pi also has 512 MB of on-board memory (RAM) which makes it possible to store more than one minute of an 8-bit input signal in the memory when sampled at 5 MHz. It was therefore hypothesized that the Raspberry Pi has the capability to record a digital output from an APD that was used for detecting a DCS signal in an optical circuit. 

Once a time-varying signal from the APD is acquired, the processor on the Raspberry Pi can also be used to compute and display the autocorrelation function of the signal or to transfer all of the sampled data to another device for processing. For the experiments shown here, the Raspberry Pi was only used to record the output of the TTL pulses from the APD to show proof-of-concept for data acquisition. The collected data was subsequently transferred (via a second Raspberry Pi) to a laptop PC running MATLAB^®^ (Mathworks, Natick, MA, USA) for processing and analysis.

## 2. Experimental Section

### 2.1. DCS Instrumentation

DCS measurements were obtained using a fiber-based system as previously described [18]. The light source was a stabilized coherent 30 mW laser diode emitting at 785 nm (Innovative Photonic Solutions, Monmouth Junction, NJ, USA) and was coupled to a 200 µm source fiber to deliver light (~100–200 µW) to the surface of interest. The diffusely reflected optical signal from the surface of interest was collected by a detector fiber (10 µm diameter) placed with center-center distance of 1.5 mm from the source fiber. The distal end of this detection fiber was coupled to a single photon silicon APD module running in the Geiger mode (ID 100-50; ID Quantique, Carouge-Genève, Switzerland) which sensed the reflected intensity signal and produced a digital output (with a maximal sampling rate of 20 MHz). This digitized readout from the APD was detected by using a commercial hardware correlator (Flex02-01D [19]) or by using the Raspberry Pi based system described below.

### 2.2. Acquisition of an Input Digital Signal Using the Raspberry Pi

The APD signal was output through a Bayonet Nut Connector (BNC) cable. The ground and signal channels from the BNC output of the APD were wired into a circuit and directly connected to two pins on the GPIO chip (pins 7 and 17) on the Raspberry Pi since the voltage output from the APD remained between 0–2 V. In order to obtain the autocorrelation of a time-varying signal using the Raspberry Pi, it is necessary to acquire the input signal at a high, fixed sampling rate (5–20 MHz) for as long a time interval as needed (0.1–5 s). A long uninterrupted acquisition of the digitized APD signal using the GPIO was not achieved using the default Linux operating system on the Raspberry Pi (due to the multi-tasking nature of the operating system). Attempts to increase the lengths of uninterrupted acquisitions using a real-time Linux kernel did not show any significant improvements. Thus, a custom operating system (OS) was written in ARM assembly language to acquire (a fixed length of) data from the TTL signals output from the APD, which was detected across two GPIO pins and then stored to memory (RAM). The custom OS did not, however, have the required functionality to store the sampled data to permanent storage. Therefore, once the full signal was acquired in memory, the custom OS transferred this signal to a second Raspberry Pi device that was running Linux, using a simple handshake protocol that was communicated through separate GPIO pins. The wiring diagrams, the GPIO handshake protocol for data transfer between the two Raspberry Pi devices, and the pseudocode for the custom OS are provided in the Appendix. The source code for the custom OS and the handshake GPIO code for the second Raspberry Pi, along with a brief user manual to help set up the two Raspberry Pi devices, have also been made available as an open-source resource online [20].

In the custom OS, we also stored the total number of clock ticks that elapsed in reading the GPIO data and storing it to memory during the acquisition of a full-length signal. These total elapsed clock ticks were also transferred to the second Raspberry Pi, along with the acquired signal TTL pulse sequence. At the time of computing the autocorrelation of the acquired signal, the total number of elapsed clock ticks for a full acquisition was divided by the length of the signal acquired to determine the number of clock ticks for each GPIO read/store instruction, which was then used to compute the time elapsed between successive reads. We found that this value remained independent of the length of a signal acquired and for all data collected here, 137 clock ticks elapsed between successive GPIO reads. Given a clock speed of 700 MHz, we calculated our custom OS achieved a sampling frequency of ~5.2 MHz (or that 0.19 µs elapsed between successive reads).

### 2.3. Software Autocorrelation

The task of the correlation device in DCS is to sample the digital signal output by the photon counter and calculate its temporal autocorrelation. In the hardware correlator, this is achieved through an organized array of electronic logic gates which allow for computation of the autocorrelation function. Since we are dealing digitized signals, the intensity output by the APD can in general be denoted as an indexed array, *Ij*, where *j* represents the time-bin corresponding to the detected time-interval, with the first bin being at *j* = 0 and the last bin being at *j* = *N* (which specifies the total collection time = *N*Δ*t*, where 1/Δ*t* is the sampling frequency). In this representation the normalized autocorrelation function can be defined using Equation (1).

(1) $$g_{2}\left(\tau \right)=\frac{1}{{\langle I\rangle }^{2}}\overset{j=N}{\underset{j=0}{\sum }}\langle I_{j}I_{j+\tau }\rangle$$

Here, *g*_2_(τ) is the normalized autocorrelation function of the signal *I* and the angular brackets represent an average. A direct algorithm to compute the autocorrelation function of a temporal sequence of signals *I_j_* is called the overlap-add method [12]. *I_j+τ_* is calculated by shifting the intensity signal by a delay time *τ*. Then, each element in the original signal *I_j_* is multiplied with the corresponding element in the shifted signal *I_j+τ_*, and *g_2_*(*τ*) is calculated by keeping a running sum of the products. A modified version of this algorithm called the multitau scheme is used by most hardware correlation devices (including the one used here) as described previously [21]. Although there is significant gain achieved in the multitau scheme by periodically reorganizing the sampled signal into a down-sampled version with a lower temporal resolution for signals measured at longer times, the overall algorithm still requires summation across every element that needs to be calculated for each value τ. This nested loop structure has performance efficiency that approaches O(*N*^2^) for *N* points.

Since the autocorrelation function is, by definition, the convolution of the intensity function with itself, this operation can be re-expressed in the Fourier domain [21] using the Wiener-Khinchin theorem as:

(2) $$g_{2}\left(\tau \right)=\frac{{ℱ}^{-1}\left(ℱ\left(I\right){ℱ}^{*}\left(I\right)\right)}{{\langle I\rangle }^{2}}$$

Here, ℱ(I) and ${ℱ}^{*}\left(I\right)$ represent the Fourier transform of the signal *I* and its complex conjugate, respectively. For a discrete signal, these transforms can be computed efficiently using the fast Fourier transform (FFT), as was recently shown by Dong *et al.* [21]. The performance of this algorithm approaches O(*N*log(*N*)), which makes it significantly faster than the overlap-add method for large *N*. As noted before, all the autocorrelation functions of the DCS data in this manuscript that were collected using the Raspberry Pi were computed using Equation (2) on a laptop computer running MATLAB^®^.

### 2.4. Pump-Controlled Flow Experiments 

Two sets of DCS experiments were performed to compare flow rates detected using the Raspberry Pi correlation device against the traditionally used hardware autocorrelation device [19]. The first set of experiments used a syringe pump that could deliver a fixed flow rate. The flow channel was a clear plastic tubing of 1 mm diameter and was connected to the flow pump at one end and to a reservoir at the other. The solution pumped through the tubing was a suspension of 1 µm diameter polystyrene microspheres (19406-15; Polysciences Inc., Warrington, PA, USA) and flow rates ranged between 0–0.140 mL/s. At each flow rate, the DCS probe was placed in contact with the flow channel with the source and detector fibers directly atop the channel and three separate measurements (each for a total collection time of 1 s) were acquired. For each acquisition, the APD output was detected both by the Raspberry Pi correlation device and the hardware correlator in order to measure the autocorrelations of the reflected signal.

### 2.5. In Vivo Cuff-Occlusion Experiments

In the second experiment, the two correlation devices were compared to each other for measuring blood flow rates through the hand of a human volunteer in a cuff-occlusion experiment. The cuff-occlusion method has previously been used to change blood flow rates *in vivo* in limbs of human subjects during DCS measurements [21,22,23]. Here, a blood pressure cuff was attached to the upper left arm of a healthy 19-year-old male volunteer in order to occlude blood flow to the hand. DCS measurements were obtained for three states: before the cuff was applied (baseline), during occlusion, and immediately after occlusion. When the pressure cuff is inflated, it constricts blood flow in the arm, and when the pressure cuff is deflated, normal blood flow is expected to be reestablished. 

As in the pump experiments, DCS probe was placed on the pad of the left thumb of the volunteer and experimental measurements were made using both correlation devices. However, in this case, experimental measurements were obtained sequentially using each device. For each state (baseline, occlusion, and post-occlusion), three consecutive DCS measurements were obtained, as in the case of the pump-flow experiments. Blood flow was occluded by inflating the cuff to a pressure of 200 mmHg (the highest tolerable pressure by the volunteer) for 15 s. The pressure was then released and immediately followed by acquisition of DCS measurements post-occlusion. We expect the blood flow measured during the occlusion to be lower than the flow measured at baseline, and that the flow measured post-occlusion to be higher than the flow observed at baseline, as reported by others [21,22,23].

### 2.6. Processing and Quantification of Acquired Autocorrelation Signals

**Figure 1:**
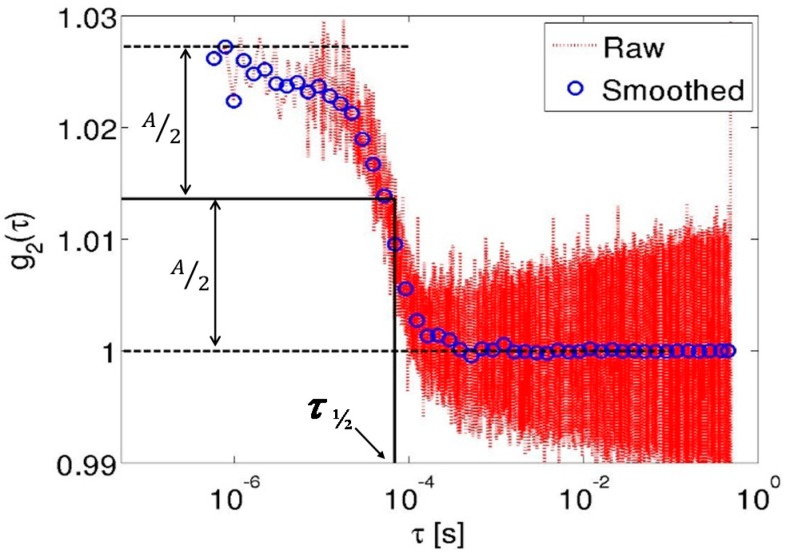
The autocorrelation curve *g*_2_(τ) for a representative flow-channel experiment calculated numerically using the Fourier transform (dotted line) from the data acquired using the Raspberry Pi and its down-sampling to logarithmically, uniformly spaced points (blue circles). The amplitude, *A*, of *g*_2_(τ), as well as the value of τ_½_, are also indicated.

Autocorrelation plots are expressed as functions of the correlation delay times (τ) and are conventionally plotted using a log-scale for the correlation times, as they spans several decades (0.1 µs^−1^·s). The collection and processing of data by the hardware correlator naturally produces logarithmically spaced delay times for the computed autocorrelation function. However, for the FFT-based algorithm, the autocorrelation necessarily needs to be computed from linearly spaced delay times. Figure 1 shows the full autocorrelation computed from a single measurement sequence of the temporal intensities using the Raspberry Pi as well as the down-sampled autocorrelation curve. The down-sampling algorithm calculated the autocorrelation at a given set of logarithmically, evenly spaced delay times (that were matched to the delay times obtained from the hardware correlator). This was done by computing the average value of the linear FFT autocorrelation data for all the linearly spaced delay times that were closest to the logarithmically spaced delay times. Figure 1 shows both the directly computed autocorrelation using the linearly spaced delay times (red dots) and the down-sampled autocorrelation at logarithmically spaced delay times (blue circles) from data collected during one acquisition of the pump flow experiments (0.14 mL/s).

The measured autocorrelation signal represents the normalized temporal intensity autocorrelation *g_2_*(τ) and is related to the normalized field autocorrelation function *g_1_*(τ) through the Siegert relationship: *g_2_*(τ) = 1 + β(*g_1_*(τ))^2^ [12,15]. In DCS, the normalized field autocorrelation *g_1_*(τ) = *G_1_*(τ)/*G_1_*(0), where *G_1_*(τ) is related to the time-varying electric field in a scattering and absorbing medium. *G_1_*(τ) is hypothesized to obey a diffusion-type equation that governs photon propagation in a turbid medium and has closed-form analytical expressions that relate *G_1_*(τ) to the mean-square displacement of a scattering particle [12,15]. The mean-square displacement, in turn, is related to the particle velocity (or flow) given the optical absorption and scattering coefficients of the medium and is extracted analytically in DCS experiments [12,15]. Since accurate measurements of the optical properties for samples used were not made in our experiments, we used an empirical model to quantify experimentally measured autocorrelations as described below.

We chose to quantify the autocorrelation curves using a derived parameter, τ_½_. τ_½_ was defined as the correlation time when *g*_2_(τ) was reduced to 50% of its full amplitude, where the full amplitude of *g*_2_(τ) is given by the difference between its highest and lowest values (see Figure 1). In general, the larger the τ_½_, the slower the flow; thus, the reciprocal of τ_½_ was used as an indicator of the measured flow rates in the remainder of this manuscript which bears out the experimental fact that autocorrelation traces decay faster for higher flow velocities.

## 3. Results and Discussion

### 3.1. Pump-Controlled Flow Experiments

**Figure 2:**
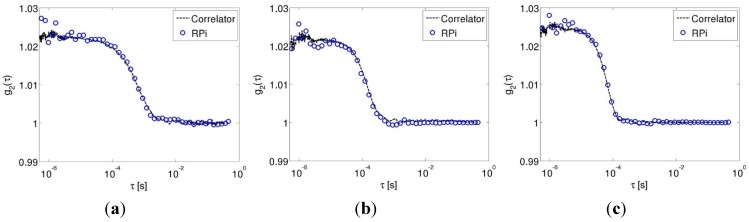
Autocorrelation traces acquired directly from the hardware correlator (line) and numerically computed from the acquired signal on the Raspberry Pi (symbols) for three different flow rates: (**a**) 0 µL/s; (**b**) 56 µL/s; (**c**) 140 µL/s.

Figure 2a–c show representative autocorrelation traces acquired using the hardware correlator and the Raspberry Pi at three different flow rates (0 µL/s, 56 µL/s and 140 µL/s), respectively. The data in each figure shows that the autocorrelation curves obtained from the data collected using the Raspberry Pi were nearly identical to those collected using the hardware correlator. It can also be seen from these figures that the autocorrelation curves decay slower (shifted to the right) for slower flow rates and decay rapidly (shifted left) for faster flow rates, as expected.

**Figure 3:**
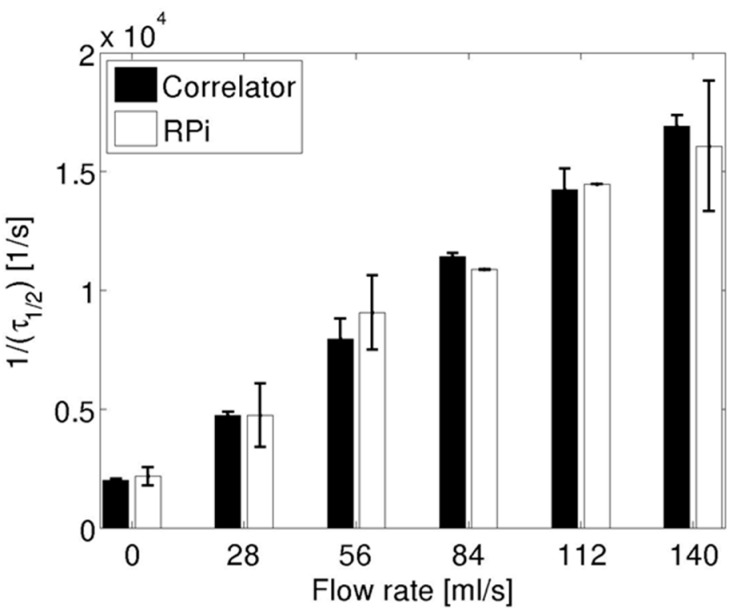
Flow parameter (1/τ_½_) for the pump flow experiments using data acquired using the hardware correlator (black bars) and Raspberry Pi (white bars). Bars represent the mean flow parameter across three repeated measurements and error bars are standard deviations.

In order to quantify the autocorrelation traces across different flow rates, they were parametrized using τ_½_ as indicated before. Figure 3 shows the reciprocal of τ_½_ for data collected using the hardware correlator (black bars) and for those collected using the Raspberry Pi (white bars). The data from both devices are consistent with the fact that the derived flow parameter increased with pump flow rates. Overall, there was less than a 15% difference in the extracted flow parameter between the two devices. However, the standard deviations were about three times larger for the data acquired on the Raspberry Pi relative to those acquired using the hardware device across all flow rates.

### 3.2. Cuff-Occlusion Experiments

**Figure 4:**
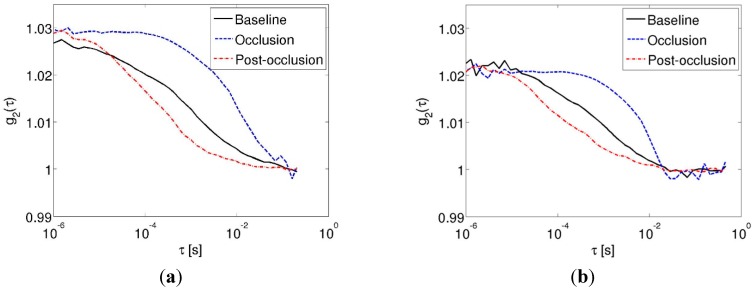
Autocorrelation traces in the cuff occlusion experiments (solid black line—at baseline; dashed blue line—during cuff occlusion; dashed-dotted red line—immediately post-occlusion) acquired using the (**a**) Hardware correlator; (**b**) The Raspberry Pi.

Figure 4 shows representative autocorrelation traces for the three conditions investigated in the *in vivo* cuff occlusion experiments. The data collected using the hardware correlator are shown in Figure 4a, while those collected using the Raspberry Pi are shown in Figure 4b. From curves shown, it can be seen that the autocorrelation trace decays more slowly during occlusion (dotted blue line) relative to baseline (black line), while the autocorrelation traces obtained immediately after cuff release (dashed-dotted red line) decays faster than the curve obtained at baseline. These trends were observed in the data obtained using either device, indicating that the fidelity of the Raspberry Pi device in regards to DCS signal acquisition was comparable to the hardware correlator, for these *in vivo* experiments.

**Figure 5:**
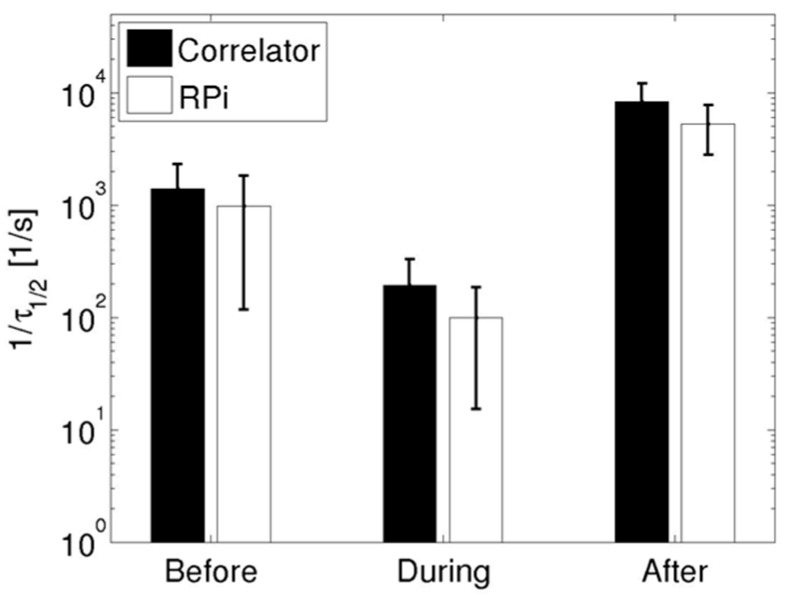
Parametrized 1/τ_½_ values before, during, and after cuff occlusion for the *in vivo* experiments for data acquired using the hardware correlator (black bars) and the Raspberry Pi (white bars). The bars indicate mean values from three repeated measurements while the error bars indicate the standard deviation.

As in the pump flow experiments, each autocorrelation trace acquired was characterized using τ_½_ to quantify the measurements. Figure 5 shows the mean 1/τ_½_ values (averaged across three acquisitions for each experimental condition) using data acquired from both devices (black bars for the hardware correlator, white bars for the Raspberry Pi device). It is to be noted that these data are plotted on a semi-log scale to capture the dynamic range exhibited by 1/τ_½_ values.

As observed in the autocorrelation traces in Figure 4, the reciprocal of τ_½_ during occlusion was lower in comparison to both baseline (before) and post-occlusion (after) values. These trends are consistent with the expected physiological response in a healthy human subjected to cuff occlusion experiments, as noted previously [21]. Data from the hardware correlator indicated that 1/τ_½_ increased (relative to the occlusion value) by factors of 7.3 and 43.7 at baseline and post-occlusion, while the increases in 1/τ_½_ measured using the Raspberry Pi were 9.8 and 53.0, respectively. In contrast to the pump-controlled flow experiments, the standard deviations for the data obtained using the Raspberry Pi were nearly equal to those obtained using the hardware device for the cuff-occlusion data.

## 4. Discussion and Conclusions

A Raspberry Pi-based digital signal acquisition device capable of sampling a TTL signal at a uniform rate of over 5 MHz was constructed. The acquired signal was then processed and filtered using a software-based technique to compute its autocorrelation function. The quality of the computed autocorrelations indicated that they were sufficiently reliable for the analysis of DCS measurements. The performance of the Raspberry Pi based device was compared to the hardware correlator in DCS experiments involving both pump-controlled flow and a human volunteer-based experiment using cuff occlusion. In both experiments, the data acquired using the Raspberry Pi device yielded results quantitatively equivalent to those acquired through a hardware correlator.

Although the technique and data presented here demonstrate a proof-of-principle potential concept where a hardware correlator device may be replaced by a Raspberry Pi device for DCS applications, there are two important hurdles that remain to be overcome before such a solution becomes practically feasible. First, the Raspberry Pi device must be shown capable of computing the autocorrelation function of an acquired raw signal, and second, the Raspberry Pi device must be able to store the collected signal (and/or its autocorrelation) in permanent storage via Universal Serial Bus (USB) or the Secure Digital card (SD). Both these tasks, though achievable, require significant effort to be made possible as they need to be programmed into the bare-metal OS running on the device. Furthermore, having a bare-metal OS capable of writing to external storage on the Raspberry Pi could impact the device’s ability to uniformly sample a given signal via the GPIO (due to interrupt handlers that might be required) and would need to be studied carefully. Once the device is shown to be able to achieve uniform sampling of a signal, the task of computing its autocorrelation can be achieved using open-source Fast Fourier Transform libraries [24]. Given that the next generation Raspberry Pi devices are expected to have faster processors and more memory, these difficulties definitely appear to be surmountable.

DCS is typically considered an expensive modality as both the light sources and correlators required can be costly. We believe that we have described a process to reduce DCS instrumentation cost by potentially substituting the hardware correlator with a low-cost Raspberry Pi minicomputer. With progress in the semiconductor display industry, it is conceivable that the cost of the light sources required will decrease as well. These advances could help the development of a low-cost DCS system, thereby increasing the adoption of DCS-based sensing, potentially opening the possibility of this technique being deployed in ambulatory and/or resource-limited settings.

## Figures and Tables

**Figure A1:**
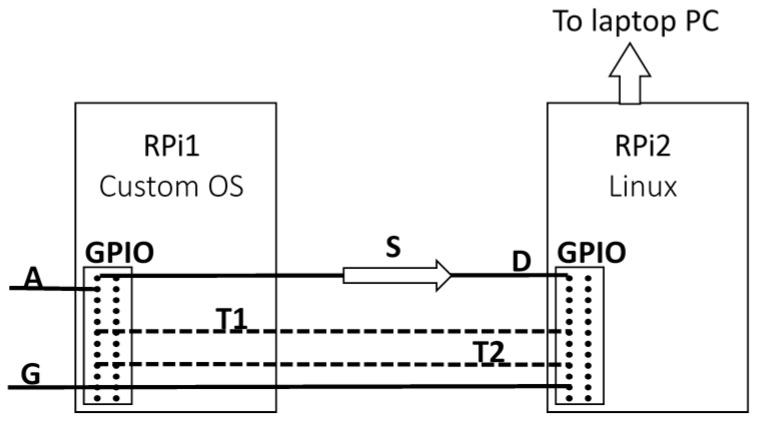
Wiring diagram schematic showing the GPIO wiring connections for the two Raspberry Pi devices. Channel A: data signal (from APD); Channel G: ground signal (from APD); Channel D: data signal; Channel T1: Rpi1 send/receive; Channel T2: Rpi2 send/receive; S: signal to be transferred.

**Figure A2:**
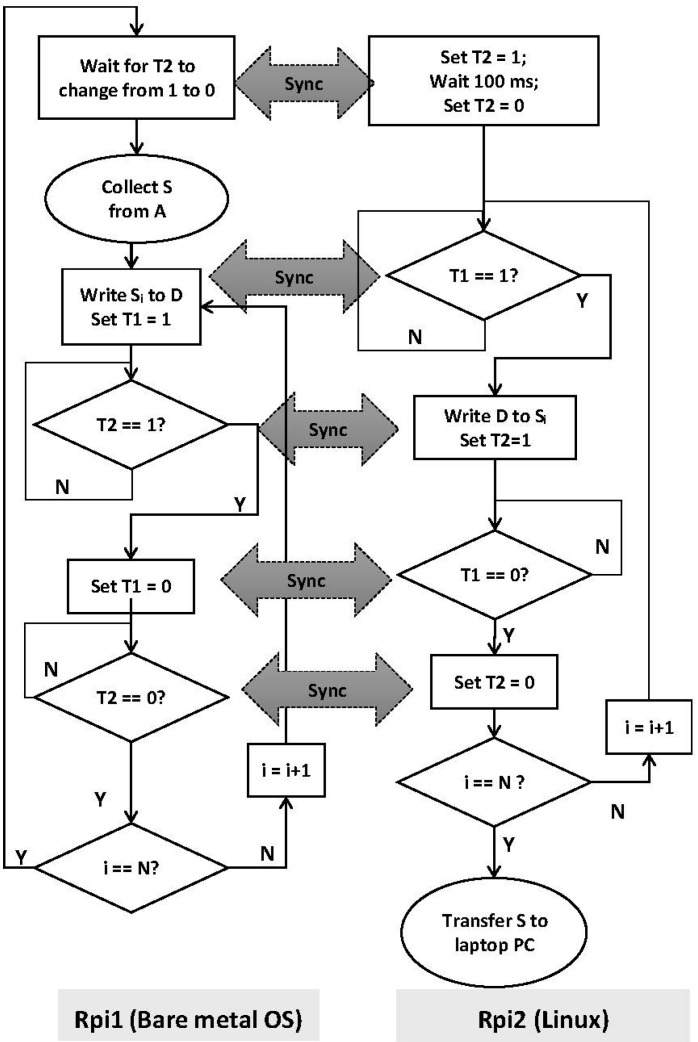
Algorithm for data transfer of the acquired signal S via GPIO between the two Raspberry Pi devices. The signal S has length N. The grey double-arrow boxes indicate the syncing of transfer in the protocol used.

## Appendix

Figure A1 shows the electrical wiring diagram between the two Raspberry Pi devices. The GPIO configuration (as per the source code provided [20]) is: A = Pin 17; G = Pin 06; D = Pin 21; T1 = Pin 24; T2 = Pin 23.

The pseudocode of the bare-metal OS showing the initialization, signal acquisition, and data transfer protocol implemented (the left half of the flowchart in Figure A2) is given below. The ARM assembly source code, a set of basic instructions for use, and the data start/stop signaling, as well as data transfer for use on the Raspberry Pi running Linux, are provided for download in Reference [20].

Initialize pins A (17) and T2 (23) as input 
Initialize pins D (22) and T1 (24) as output

Initialize CPU cycle counter

**main:**
        **wait1_for_rpi2:** 
        if (pin T2 is LOW) {go to **wait1_for_rpi2**}
        **wait2_for_rpi2:**
        if (pin T2 is HIGH) {go to **wait2_for_rpi2**}
        ti = read CPU cycle counter
        i = 1
        **collect_signal:** 
                  read GPIO state and store to S[i]
                  i = i + 1
        if (i < N + 1) {go to **collect_signal**}
        tf = read CPU cycle counter	
        store (tf – ti) to S[0]
        i = 0
        j = 0
  **transfer:**
                if (i == 0) {
                         d = jth bit of S[0]
                         j = j + 1
                         if (j == 32) {i = i + 1}
                }
                if (i > 0) {
                             d = Value of Pin A from S[i] 
                             i = i + 1
                }
                set pin D to 0
                if (d = 1) {set pin D to 1}
                set T1 to HIGH
          **handshake1:**
                if (T2 = LOW) {go to **handshake1**}
                set T1 to LOW
          **handshake2:**
                if (T2 = HIGH) {go to **handshake2**}
        if (i < N) {go to **transfer**}
if {i = N} {go to **main**}
